# Beyond the Liver: Liver-Eye Communication in Clinical and Experimental Aspects

**DOI:** 10.3389/fmolb.2021.823277

**Published:** 2021-12-24

**Authors:** Tian-Hao Yuan, Zhen-Sheng Yue, Guo-Heng Zhang, Lin Wang, Guo-Rui Dou

**Affiliations:** ^1^ Department of Ophthalmology, Eye Institute of Chinese PLA, Xijing Hospital, Fourth Military Medical University, Xi’an, China; ^2^ Department of The Cadet Team 6 of School of Basic Medicine, Fourth Military Medical University, Xi’an, China; ^3^ Department of Hepatobiliary Surgery, Xijing Hospital, Fourth Military Medical University, Xi’an, China

**Keywords:** liver-eye communication, metabolism, non-alcoholic fatty liver disease, diabetic retinopathy, traditional Chinese medicine, communication molecule

## Abstract

The communication between organs participates in the regulation of body homeostasis under physiological conditions and the progression and adaptation of diseases under pathological conditions. The communication between the liver and the eyes has been received more and more attention. In this review, we summarized some molecular mediators that can reflect the relationship between the liver and the eye, and then extended the metabolic relationship between the liver and the eye. We also summarized some typical diseases and phenotypes that have been able to reflect the liver-eye connection in the clinic, especially non-alcoholic fatty liver disease (NAFLD) and diabetic retinopathy (DR). The close connection between the liver and the eye is reflected through multiple pathways such as metabolism, oxidative stress, and inflammation. In addition, we presented the connection between the liver and the eye in traditional Chinese medicine, and introduced the fact that artificial intelligence may use the close connection between the liver and the eye to help us solve some practical clinical problems. Paying attention to liver-eye communication will help us have a deeper and more comprehensive understanding of certain communication between liver diseases and eyes, and provide new ideas for their potential therapeutic strategy.

## Introduction

In recent years, the communication between organs has received more and more attention. With the development of modern medical physiology and pathology, it has been discovered that there are some communication links between the human body’s organs and organs or tissues that are established with the help of endocrine, immune and other systems, which are considered to be important for maintaining homeostasis and achieving physiological functions. The connection between the eyes and the liver has been discovered and valued in many studies (Wang et al., 2021), which is achieved through a variety of pathways including metabolism, inflammation, oxidative stress, and immunity. In addition, clinically, some possible connections have been revealed in the occurrence, development and outcome of some liver diseases and ocular diseases, such as Non-alcoholic fatty liver disease (NAFLD) and Diabetic retinopathy (DR). Therefore, if we have a clearer understanding of the communication mechanism between the liver and the eyes, it will help us better understand the development mechanism of liver and eye diseases, and provide some ideas for targeted therapy.

## Communicating Molecules Mediate the Liver-Eye Association

**FIGURE 1:**
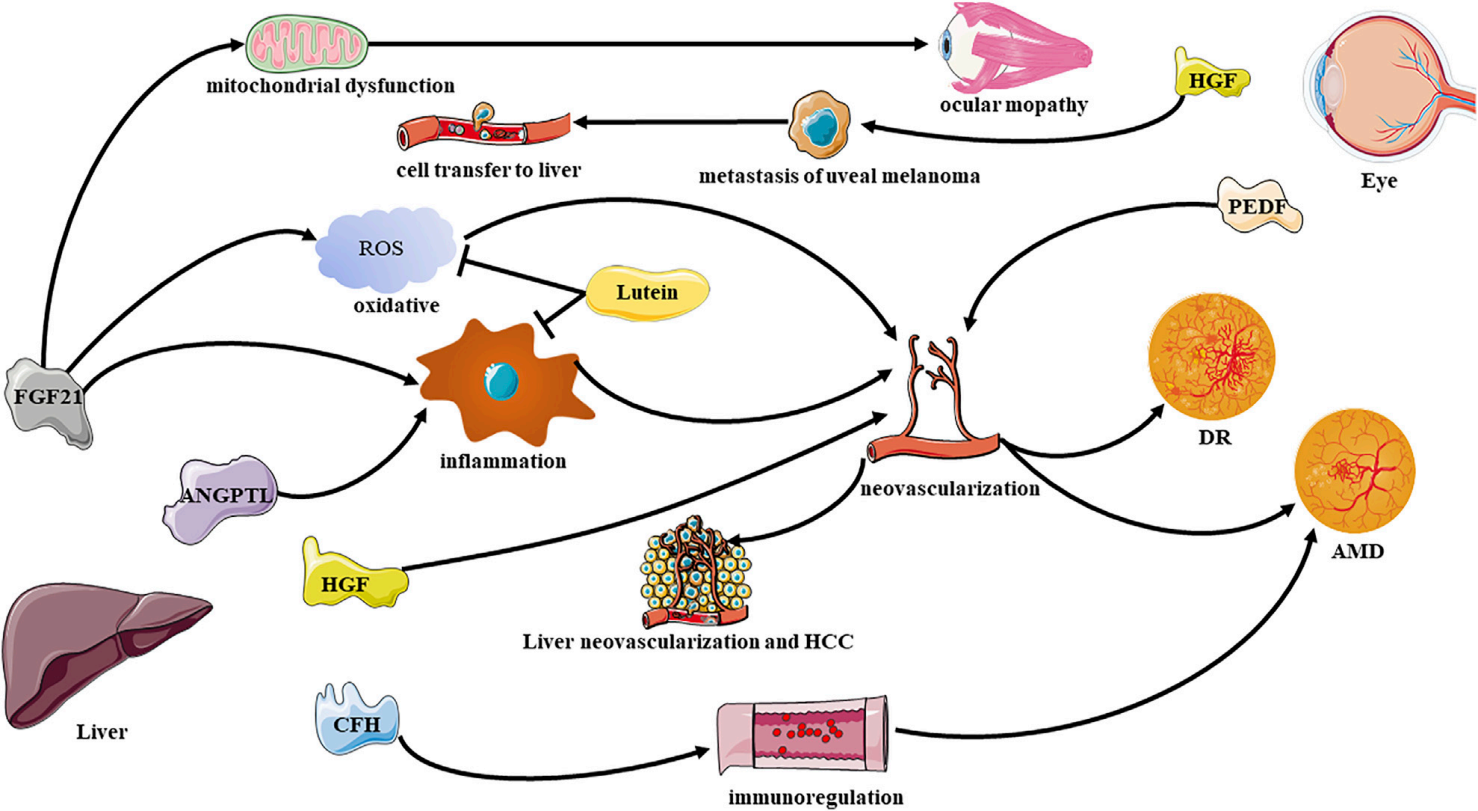
Effective communication molecules from the liver to eye under physiological and pathological conditions. DR, diabetic retinopathy; AMD, age related macular degeneration; FGF21, Fibroblast Growth Factor 21; HGF, hepatocyte growth factor; ANGPTL, Angiopoietin-like proteins; PEDF, pigment epithelium-derived factor.

The interaction between the liver and the eye is reflected in the molecular communication by their secretory factors and their associated cytokines (Figure 1). “Hepatokines” are certain signaling proteins that are secreted exclusively or predominantly by the liver (Wang et al., 2021),which are mostly delivered to liver or other distant organs through the human circulation system, and are involved in regulating diseases such as metabolic, inflammatory disease (Meex and Watt, 2017). Similarly, some factors secreted by the eyes also remotely affect the state of the liver. In addition, some non-organ-specific cytokines also reflect the connection between the liver and the eyes.

### Fibroblast Growth Factor-21

Fibroblast growth factor-21 (FGF-21) is a hormone predominantly secreted by the liver, which could perform multiple effects on the regulation of glucose metabolism as well as insulin activity (Xu et al., 2009; Markan et al., 2014). The function of FGF-21 to regulate glucose homeostasis has been universally proven in animals (Potthoff et al., 2012) and humans (Gaich et al., 2013). In addition, FGF21 is also defined as highly predictive biomarker for mitochondrial diseases (Tsygankova et al., 2019). FGF21 has been found in clinical research to reflect the liver-eye connection in many aspects. A study has found that FGF21 is significantly related to ocular myopathy (a mitochondrial disease), especially chronic progressive external ophthalmoplegia (Morovat et al., 2017). Moreover, FGF21 can also affect autophagy, the level of FGF21 increases under fasting induction, which can dephosphorylate the transcription factor EB, in addition induce the expression of genes related to autophagy (Chen et al., 2017a). Autophagy defects, including lipofuscin accumulation, decreased mitochondrial activity, and elevated reactive oxygen levels, can affect angiogenesis. Mutations of autophagy genes and the occurrence of autophagy defects are considered to be related to the occurrence of age related macular degeneration (AMD) in animals (Zhang et al., 2017a) and humans (Golestaneh et al., 2017). FGF21 administration decreased neovascular lesions in two models of neovascular age-related macular degeneration has been observed recently (Fu et al., 2017). Oral peroxisome proliferator-activated receptors-alpha (PPARα) agonists can be used in insulin-deficient diabetic mice (Fu et al., 2018), intraperitoneal injection of streptozotocin to induce diabetes model mice (Tomita et al., 2020a), oxygen-induced retinopathy model mice (Tomita et al., 2019), and retinal ischemia model mice (Lee et al., 2021) to promote the expression of FGF21.

The production of FGF21 is induced by PPAR-α and plays a role by regulating the activities of PPAR and PGC-1α (Potthoff et al., 2009). A research has shown that FGF21 transcription cannot be induced in the liver of PPAR-α deficient mice (Lundåsen et al., 2007). The increase in FGF21 expression level boosting liver function, maintain retinal neuron activity, regulating pathological microglia proliferation, strengthening the retinal antioxidant defense system, reducing pro-inflammatory cytokines and improving retinal function, and mediate and inhibit retinal neovascularization. Some recent basic medical studies have proved that FGF21 deficiency can lead to aggravation of retinal neovascularization. FGF21 may inhibit retinal neovascularization by inhibit the expression of tumor necrosis factor (TNF)-α and increasing the secretion of adiponectin (Lin et al., 2013; Fu et al., 2017), indicating that FGF21 may have therapeutic significance for DR. But it is worth noting that it has been reported that serum FGF21 concentration is positively correlated with the severity of DR (Lin et al., 2014), this seems to contradict the previous conclusion. The increased serum FGF21may be related to the compensation caused by FGF21 resistance, suggesting that FGF21 as a potential biomarker of DR. What’s more, the analogues of long-acting FGF21 have recently been found to improve the permeability of tight junctions by increasing the level of tight junction proteins for example Claudin-1 in human vascular endothelial growth factor (VEGF)-induced human retinal microvascular endothelial cells and C57BL/6J mice, leading to the reduction of vascular leakage in retinal diseases (Tomita et al., 2020b). All in all, FGF21 has an effect on Pterygia (Yaghoobi et al., 2020), AMD, DR and many other eye diseases because of its ability to reduce ocular neovascularization. Therefore, it seems promising to use FGF21 as a treatment direction for ocular vascular diseases.

### Hepatocyte Growth Factor

Hepatocyte growth factor (HGF) is a cytokine mainly secreted by kupffer cells of the liver, but the expression of HGF receptors has been detected in the cornea, lens and retinal tissues of the eye, which can maintain the structure and function of corneal epithelial cells, lens epithelial cells, and retinal pigment epithelial cells (Grierson et al., 2000). Uveal melanoma is the most common primary intraocular malignant tumor, and 50% of patients eventually die of metastatic disease. The most common site of metastasis is the liver (Shields et al., 1991). HGF can play a role in promoting cell transfer. The activation of PI3K/AKT pathway induced by HGF-cMET axis participates in the down-regulation of cell adhesion molecules E-cadherin and β-catenin, which weakens cell adhesion and promotes the induction of tumor cell proliferation, movement, adhesion and invasion (Ye et al., 2008). Besides, HGF also has an effect on retinal neovascularization. HGF/NK-4 inhibits VEGF induced retinal angiogenesis by inhibiting the phosphorylation of ERK and ETS-1 in endothelial cells cultured *in vitro* and rabbits (Nakabayashi et al., 2003). Moreover, retinal pigment epithelial cells (RPE)-endothelial-mesenchymal transition is related to a variety of blinding retinal diseases. In the study of endothelial-mesenchymal transition induction on the RPE layer derived from human induced pluripotent stem cells, it was found that the HGF-MET signal showed the highest overall enrichment. In addition, they also found that HGF signaling plays a role in regulating the transcription profile of RPE (Mertz et al., 2021).Thus, it will be intriguing to study whether altered expression of HGF in liver diseases could exsert distance influence on ocular condition.

### Angiopoietin-Like Proteins

Angiopoietin-like proteins 4 (ANGPTL-4) and ANGPTL-8 are a class of proteins mainly secreted by the liver, and a very small part is produced by adipose tissue and muscle (Wang et al., 2021). ANGPTL4 transcript in adipose tissue accounts for only 10% of liver in human (Romeo et al., 2009). Therefore, we have reason to believe that ANGPTL-4 in the human circulation mainly comes from the liver. The detection of protein levels in HRMEC cultured *in vitro* and rat retinal and vascular endothelial cell extracts showed that high glucose can induce the up-regulation of ANGPTL-4 expression in both models, and it may increase the expression of ANGPTL-4 by activating profilin-1 signal to generate retinal inflammation, vascular permeability, and angiogenesis (Lu et al., 2018). In the test of proliferative diabetic retinopathy (PDR) patients, it was found that the levels of ANGPTL-4 in the vitreous and serum of PDR patients were higher than those in the control group, and the expression level of VEGF was positively correlated with ANGPTL-4 (Lu et al., 2016). In addition, inflammatory factors such as interleukin (IL)-8 are also positively correlated with ANGPTL-4 levels (Wu et al., 2021). This suggests that ANGPTL-4 may promote angiogenesis in humans, and may increase retinal inflammation, thereby increasing the severity of PDR. ANGPTL-8 plays an important role in regulating lipid metabolism inside and outside EC, lipoprotein lipase activity, and inflammatory pathway NF-κB signal transduction (Abu-Farha et al., 2020). Similar to ANGPTL-4, the expression level of ANGPTL-8 was also detected in PDR patients, and it was significantly positively correlated with VEGF (Lu et al., 2017). ANGPTL reflects the tight connection between the liver and the eye in the inflammation pathway.

### Complement Factor H

Complement factor H (CFH) is an essential component for the synthesis of alternative pathways of complement. It is generally believed that CFH is mainly produced in the liver in the human body (Mandal and Ayyagari, 2006), and is still controversial that whether it is expressed in the retina and RPE and choroid (Hughes et al., 2016). Importantly, the correlation between AMD and CFH has been widely concerned (Klein et al., 2005). Based on blood analysis of patients with AMD, it is found that the expression level of CFHR-4 gene, which is specifically expressed in the liver, is increased in the blood and retina. The activation of this gene will activate the complement system and exacerbate the course of the disease (Cipriani et al., 2020), implicating that the liver plays an important role in regulating the immune homeostasis of the retina. In addition, Y402H variant of CFH gene proved to be associated with increased risk of AMD (Hughes et al., 2016). CFH regulates alternative pathways of complement activation and protects host cells from inappropriate complement activation (Mandal and Ayyagari, 2006). In the C57BL/6 mouse model, the expression of CFH in RPE and choroid helps to regulate the alternative pathway of complement cascade and membrane attack complex formation, thereby preventing the occurrence of choroidal neovascularization (CNV). In addition, local inhibition of CFH also weakened the regulation of membrane attack complex deposition, causing the disorder of membrane attack complex deposition, and aggravated the laser-induced CNV in mice (Lyzogubov et al., 2010). Further research is needed to explain how CFH mediates liver-eye contact through the immune system.

### Retinal Pigment Epithelium-Derived Factor

Secretory factors produced in eyes can also act on the liver. Retinal pigment epithelium-derived factor (PEDF) was initially discovered to be secreted by retinal pigment epithelial cells (Tombran-Tink and Johnson, 1989). It is a neurotrophic factor with anti-oxidation, anti-inflammatory and anti-angiogenic effects (Elahy et al., 2014). PEDF also inhibits Wnt coreceptors and low-density lipoprotein receptor-related protein 6 (LRP6) in the eyes and liver (Protiva et al., 2015), suggesting that it may play a role in liver-eye communication. It has been widely demonstrated that PEDF and VEGF together maintain the balance of controlling angiogenesis in the eye. For example, mTORC1 signal in DR can change the proliferation and migration of endothelial cells by regulating the expression of VEGF and PEDF protein (Liu et al., 2020). Its possible mechanism of action is related to inflammation-related pathways. A study has found that PEDF can inhibit angiogenesis from endothelial cells and tumor cells by down-regulating HIF-1α in breast cancer (Mao et al., 2020). Notably, PEDF is up-regulated in the liver of cirrhotic humans and bile duct-ligated rats, and the adenovirus-mediated gene transfer in bile duct-ligated rats exogenously overexpresses PEDF, which inhibits liver angiogenesis, fibrogenesis and reduces portal pressure (Mejias et al., 2015). Interestingly, the inhibitory effect of PEDF on angiogenesis is only for pathological angiogenesis, and does not affect physiological angiogenesis, which suggests the potential of PEDF for possible therapeutic applications. In hepatocellular carcinoma (HCC), PEDF can play an anti-angiogenic effect in this typical tumor (Matsumoto et al., 2004). It can also regulate epithelial mesenchymal transition by up-regulating the expression of E-cadherin and down-regulating the expression of Slug and Vimentin, thereby reducing the migration and invasion ability of HCC cells (Chen et al., 2017b). However, it is still unveiled that if the upregulated PEDF is derived from remote delivery or from local production. There have been some researches related to knockout PEDF, but no study has specific knockout PEDF in the eye. Recent research has found that the PEDF signal of the eye in the RAD6B-deficient group changes, which leads to the occurrence of retinal degeneration (Ye et al., 2022). This may provide ideas for ocular knockout PEDF and further study the source of up-regulated PEDF expression.

### Other Molecules

In addition to the oxidative stress and inflammation links between the liver and the eyes, some non-organ-specific molecules also act as mediators. Lutein is one of the carotenoids, occurs in a large green vegetables and plasma, eye of body. It has specific biological functions, especially in several ocular diseases like age-related macular degeneration (Heesterbeek et al., 2020). Mechanically, lutein can reduce light-induced oxidative damage and prevents inflammation (Li et al., 2020). Indeed, lutein supplementation improves the oxidative stress in the liver and eyes of guinea pigs on a high-cholesterol diet by reducing the binding activity of NF-κB DNA and the level of inflammatory factor TNF-α (Kim et al., 2012). Except lutein, a study showed that thioacetamide can attack the liver to secrete the pro-inflammatory cytokines IL-6 and TNF-α, which ultimately leads to brain and eye damage. More interestingly, the improvement of liver damage can improve the eyesight and cognitive ability of mice (Sun et al., 2020). It can be seen that the liver and eyes are inextricably linked with oxidative stress and inflammation.

## Liver Metabolism Affects Ocular Diseases

**FIGURE 2:**
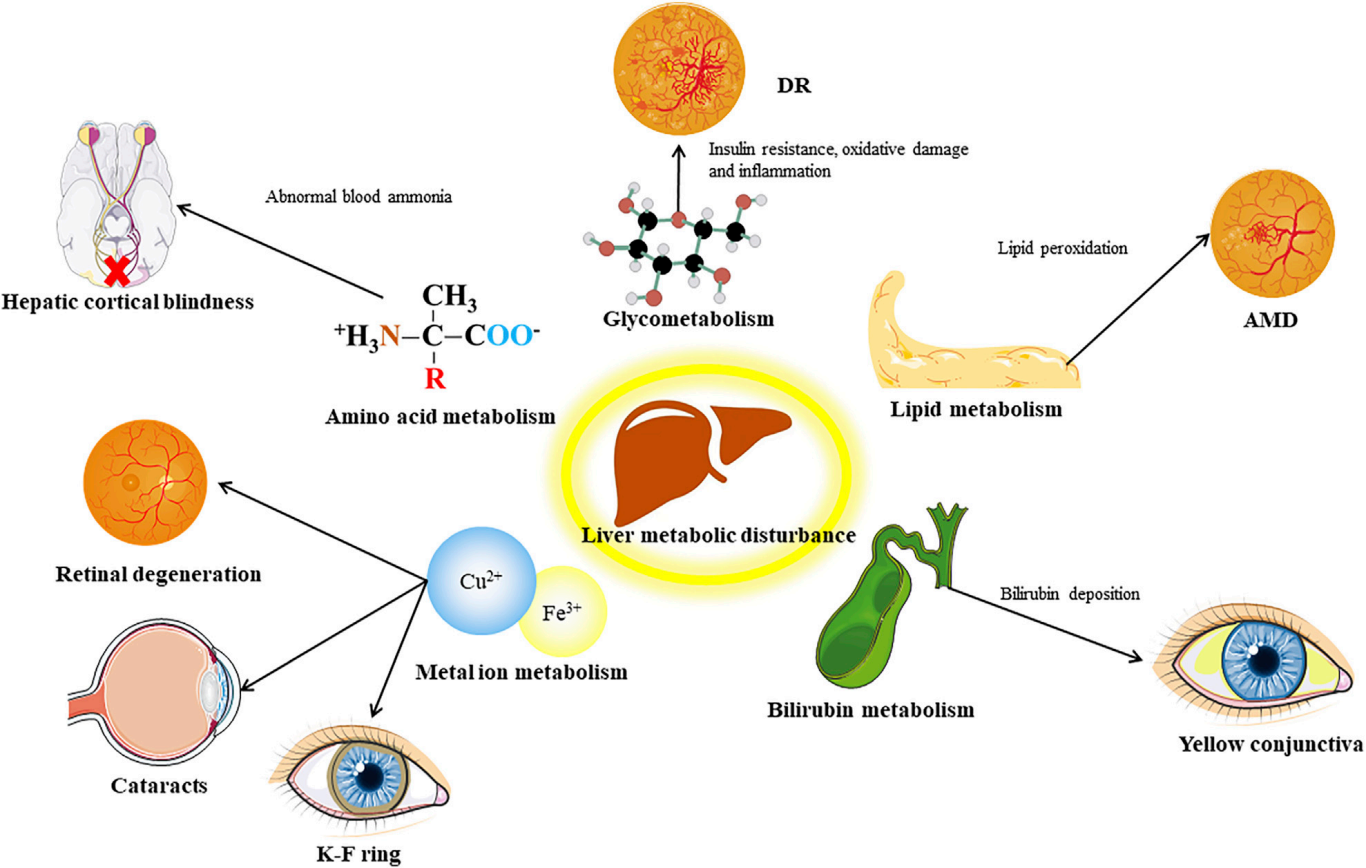
Distinct communicating by metabolic factors between liver and eye. DR, diabetic retinopathy; AMD, age related macular degeneration.

The liver is regarded as the center of metabolism in the human body, and it metabolizes carbohydrate, lipids, proteins, and many other substances. Thus, disorders of liver metabolism often influence a variety of physiological processes, and may lead to corresponding eye diseases (Figure 2).

### Glycometabolism

The liver is an important place for the body to synthesize and store glycogen, and it plays an important role in blood sugar regulation. Abnormal liver function, such as the accumulation of fat in the liver caused by NAFLD, can induce insulin resistance and increased liver gluconeogenesis and other blood glucose regulation disorders, which induce or aggravate diabetes (Roden and Shulman, 2019; Watt et al., 2019). Diabetes can cause abnormal metabolism of vascular endothelial cells in the eye and induce DR (Li et al., 2019). It is generally recognized that there is a link between NAFLD and diabetes. For example, in an 11-year follow-up study, NAFLD was found to be a risk factor for diabetes and metabolic syndrome (Adams et al., 2009). However, the connection between NAFLD and DR is still controversial, and we will discuss it later. Regulating sugar metabolism seems to be a good target for retinal neovascular diseases. PFKFB3 is a key regulatory enzyme in the glycolysis pathway (Zhou et al., 2021). PFKFB3 inhibits endothelial cells *in vitro* and damages the sprouting of EC, and affects the growth and branching of blood vessels in the mouse retina *in vivo* (Xu et al., 2014).

### Lipid Metabolism

Secondly, the liver participates in the lipid cycle by participating in the synthesis of fatty acids and lipoproteins, and plays a pivotal role in lipid metabolism (Nguyen et al., 2008). Lipid metabolism plays an important role in the pathogenesis of eye diseases, especially ocular neovascular disease. Using etomoxir to inhibit the oxidation of fatty acids in retinopathy of prematurity model mice can reduce retinal neovascularization (Schoors et al., 2015). AMD has been found to be related to a variety of lipids and lipoprotein genes, including liver lipase, cholesterol ester transferase, and apolipoprotein E, which are mainly expressed in the liver in the human body (Jun et al., 2019). Disturbance of lipid metabolism is an important pathological mechanism leading to AMD. Under oxidative stress, a type of lipid deposit called drusen is formed in the retina, and it activates the complement system to trigger chronic inflammation (Xu et al., 2018). Studies have shown that lipid imbalance can promote the development of lesions in a variety of different animal models of AMD (Malek et al., 2005; Fujihara et al., 2009; Toomey et al., 2015). Epidemiological investigations suggest that high-fat diet is a risk factor for AMD (Clemons et al., 2005). These evidences suggest that lipid metabolism is closely related to AMD, and is related to the liver function.

### Amino Acid Metabolism

Another important biological function of the liver is to deaminate and transaminate amino acids and use the ammonia produced by amino acid metabolism to synthesize urea to prevent excessive levels of ammonia in the blood (Walker, 2014). When liver disease occurs, it will affect the normal clearance of ammonia in the blood, which will have a toxic effect on the optic nerve and cause hepatic cortical blindness (Ammar et al., 2003).

### Bilirubin Metabolism

The liver also plays an important role in the metabolism of bilirubin. Liver dysfunction caused by liver disease can cause liver cells to fail to normally take in unbound bilirubin in the blood, causing bilirubin metabolism disorders in the body. Elevated bilirubin can cause yellowing of the skin and conjunctiva, especially the conjunctiva. Because the conjunctiva contains more elastin, it has a higher affinity with bilirubin (Carroll et al., 2017). Yellowing of the conjunctiva in patients with jaundice is an extremely intuitive manifestation of the liver-eye connection. What’s more, primary biliary cirrhosis caused by bilirubin deposition can lead to the occurrence of pigmented corneal rings (Fleming et al., 1977).

### Metal Ion Metabolism

Moreover, liver also regulates the metabolism of some metal ions. The liver secretes hepcidin into the blood to reduce blood iron levels. The blood and retinal pigment epithelium iron levels of liver-specific hepcidin knock-out mice increase, and the free iron levels in the retina increase and cause RPE hypertrophy, the photoreceptors also undergo focal degeneration (Baumann et al., 2019). The pathogenic variants of the disease-causing genes of hepatolenticular degeneration led to the functional defect or loss of ATPase, which causes the biliary tract copper excretion disorder and leads to abnormal copper metabolism. This leads to copper deposits in the Descemet membrane area of the cornea, triggering a characteristic lesion called the Kayser–Fleischer ring (Richard and Friendly, 1983). In addition, copper deposits in the eye can also cause ocular lesions called sunflower cataracts (Fahnehjelm et al., 2011).

## Association of Non-Alcoholic Fatty Liver Disease and Retinopathy Implies the Liver-Eye Communication

NAFLD is the umbrella term for non-alcoholic simple fatty liver, non-alcoholic steatohepatitis, and hepatic cirrhosis (Farrell and Larter, 2006) and have become the leading cause of chronic liver disease in Western countries (Lazo and Clark, 2008). It has a diverse histopathological spectrum ranging from simple steatosis with mild inflammation to various stages of fibrosis, and ultimately to hepatic cirrhosis, HCC. Previous studies have found insulin resistance is a critical factor in the pathophysiology of NAFLD and can promote the accumulation of triglycerides in the liver (Zelber-Sagi et al., 2018; Abdelmoemen et al., 2019). NAFLD can cause disorders of glucose and lipid metabolism in the body, coupled with its characteristic of insulin resistance, naturally remind people of diabetes. DR is the most common chronic complication of diabetes mellitus and one of the main causes of acquired blindness in the world (Campos et al., 2017). Recognized main pathogenic mechanism of diabetic retinopathy is hyperglycemia-induced microvascular damage caused by impaired insulin action due to insulin resistance (type 2 diabetes mellitus) or insulin deficiency (type 1 diabetes mellitus) (Gardner et al., 2011), while the deep pathogenesis of DR has not yet been fully understood (Zhang et al., 2017b).

Recently, multiple clinical investigations designed to explore the effects of non-alcoholic fatty liver disease on the incidence of DR in patients with diabetes mellitus (Song et al., 2021). There also have been more and more studies have indicated that NAFLD can influence the morbidity of complications in patients of diabetes mellitus, especially microvascular complications (Hazlehurst et al., 2016; Perumpail et al., 2017), which involve chronic kidney disease and DR (Mima, 2016). Although NAFLD and DR have some similar pathogenic and molecular mechanisms (Potthoff et al., 2009; Tomita et al., 2019; Lee et al., 2021), studies on different ethnic groups have shown different results. For type 1 diabetics, a previous study showed that the prevalence of DR increased in Indian patients with nonalcoholic fatty liver disease (Vendhan et al., 2014). Our recent meta-analysis including Indian and Japanese patients with type 1 diabetes mellitus also suggested the same conclusion (unpublished). However, for type 2 diabetics, several evidence-based medicine studies from different countries reflected that NAFLD may not be a risk factor for DR and may even be beneficial. A study that mainly includes American type 2 diabetics indicated that NAFLD is not associated with retinopathy (Lin et al., 2016). Whereas several observational studies in China, Korea and Iran showed that the NAFLD group had lower retinopathy (mainly NPDR) morbidity than the non-NAFLD group in patients with type 2 diabetes mellitus (Lv et al., 2013; Kim et al., 2014; Afarideh et al., 2019; Zhang et al., 2019; Wen et al., 2021). Additionally, in an Italian research, the NAFLD was positive related with retinopathy (NPDR or PDR) (Targher et al., 2008). Some Western studies on NAFLD patients have also shown that the more severe the liver fibrosis, the higher the risk of retinopathy (Leite et al., 2021; Mikolasevic et al., 2021). These controversial opinions can be caused by different diabetic pathological characteristics of different races (Song et al., 2021). For example, the serum insulin level of Asians was lower than Caucasian (Yoon et al., 2006; Unnikrishnan et al., 2017). Regarding the connection between NAFLD and DR, the conclusions drawn by basic medical research are also controversial. As mentioned above, FGF21 plays a role in inhibiting retinal neovascularization As a regulatory secretion mainly produced by the liver (Geng et al., 2020; Keuper et al., 2020), oxidative damage and chronic inflammation of NAFLD suppressed β-klotho and FGFR expression, leading to a compensatory increase in FGF21 synthesis and secretion (Tucker et al., 2019). It has also been proved that the level of serum FGF21 in the NAFLD group is higher than control group (He et al., 2017; Keuper et al., 2020). This seems to support the conclusion that NAFLD is negatively correlated with DR, as the increased level of FGF21 may be the reason of the lower morbidity of retinopathy in patients with NAFLD after compromising with FGF21 resistance. But for retinal artery damage, in the study by Wen et al., patients with NAFLD had higher incidences of coronary artery disease and retinal artery lesion (Yang et al., 2015), the opposite conclusion was reached. However, whether FGF21 resistance also occurs in the eyes still needs to be measure. If FGF21 resistance also occurs in the eyes, then the increase in serum FGF21 does not prove that NAFLD and DR are negatively correlated. What’s more, we should note that the current research on FGF21 inhibiting retinal neovascularization is through the administration of exogenous FGF21 or FGF21 receptor agonists (Tomita et al., 2019; Tomita et al., 2020a; Lee et al., 2021). At present, there is no research on whether the compensatory increase of FGF21 in serum in NAFLD is sufficient to cause the therapeutic effect of ocular neovascularization. Therefore, the compensatory elevated FGF21 level in NAFLD patients is not enough to indicate that NAFLD is negatively correlated with DR. What’s more it is worth noting that in a study, the use of exogenous FGF21 inhibited the occurrence of choroidal neovascularization in mice unrelated to the occurrence of diabetes (Fu et al., 2017), which suggested that there may be more communication pathways between our liver and eyes, not just only insulin resistance.

In addition, the occurrence of retinopathy might also affect the physiological process and pathological progress of the liver. Oxidative stress in the retina induced by retinitis pigmentosa might affect soluble macromolecules in retina or damage the melanopsin system. It led to chronic circadian desynchronization, weakened the antioxidant defense of the system, and eventually led to oxidative stress in the liver (Perdices et al., 2018), which may promote NAFLD. Diabetic retinopathy was also considered as a risk factor for HCC in NAFLD patients (Azuma et al., 2019). However, the exact mechanism is still to be elucidated. Therefore, patients with NAFLD complicated with diabetic retinopathy may should be regularly screened for HCC.

In all, molecular mediators linking NAFLD with diabetic retinopathy might include an increased release of some pathogenic mediators from the liver, such as FGF21 (Fu et al., 2017), HGF (Nakabayashi et al., 2003), C-reaction protein, reactive oxygen species, IL-6 and TNF-α (Targher et al., 2008), which in turn determine the disease progression, forming eye-liver communication. However, the evidence for an association between NAFLD and diabetic retinopathy is still unclear because of the tangled association between NAFLD and hyperglycemia, insulin resistance, obesity and other traditional risk factors for diabetic retinopathy and the small study population in the published literature. More researchers are needed to elucidate the correlation and underlying molecular mechanism between NAFLD and diabetic retinopathy in diabetes.

## Current Application of Liver-Eye Communication in Clinical Practice

### Traditional Chinese Medicine

A long time ago, the traditional viscera theory of traditional Chinese medicine (TCM) put forward the saying that “liver resuscitation in the eyes.” With the development of modern medicine, more and more evidences show that the two major organs of the liver and the eyes are complex and intimate in terms of physiology and pathology. The biological connection further supports the theory of “liver resuscitation in the eyes.”

Starting from the liver to treat eye diseases, it has been widely clinical practice in TCM. According to TCM differentiation, a study have analyzed the types of patients with dry eye disease, and concluded that the liver and kidney be feeble occurs most frequently among different types, which is much higher than other types (XZ et al., 2015). Therefore, in clinical practice, some scholars treat dry eye by nourishing the liver and kidney. They choose Qi-Ju-Di-Huang-Van in treatment, which promotes the secretion of tears, prolongs the tear film rupture time, reduces dryness and recurrence rate (D and WP, 2021). Pestle therapy, which is beneficial to the liver and kidney, also promotes tear secretion, prolongs tear film rupture time, and relieves the patient’s anxiety and depression (YX et al., 2020). In addition, TCM believes that the liver is the key to the treatment of inflammatory diseases. Based on this theory, people use Long-Dan-Xie-Gan-Tang, which hepatoprotective and anti-inflammatory effects, to treat uveitis (HS et al., 2015). In addition, based on the cognition of liver-eye connection, use Xiao-Yao-San, which has many benefits for the liver, to treat supraorbital neuralgia, eyeball pain, dry eye, open-angle glaucoma and achieved certain effects (J et al., 2017). We have summarized the main ingredients and effects of Chinese medicines mentioned above (Table 1). In addition, some of the ingredients of these Chinese medicines have been reported to have liver toxicity, including Alisma, licorice, and bupleurum (Frenzel and Teschke, 2016). These Chinese medicines are also not recommended for long-term use in clinical practice, so the safety of their long-term use should be paid attention to when using this type of medicine for treatment.

**TABLE 1:** The actions and possible mechanism of TCM drugs revealing liver-eye connection.

Drug name	Pharmaceutical ingredients	The effects of drug
Qi-Ju-Di-Huang-Van	*Lycium barbarum*, *Chrysanthemum*, *Rehmannia*, *Cornus*, *Peony bark*, *Chinese yam*, *Tuckahoe*, *Alisma*	Nourishes the kidney and liver. Treatment liver and kidney yin deficiency, dizziness, tinnitus, photophobia, tears in the wind, dim vision.
Long-Dan-Xie-Gan-Tang	*Gentian*, *Gardenia*, *Scutellaria baicalensis*, *Mutong*, *Alisma*, *Plantain seed*, *Bupleurum*, *Liquorice*, *Angelica*, Radix *Rehmanniae* recen	Reduce liver and gallbladder fire, clear scorching damp heat.
Xiao-Yao-San	*Licorice*, *Angelica*, *Poria cocos*, *Paeonia alba*, *Atractylodes macrocephala*, *Bupleurum*	Soothing liver and relieving depression, nourishing blood and strengthening spleen.

The therapeutic mechanism of TCM for eye diseases through the liver has also been extensively explained under the development of modern medicine. TCM believes that eating animal liver has the effect of improving eyesight. From the perspective of modern medicine, it is because the liver stores and transports vitamin A, and eating liver of animal supplements vitamin A. Lack of vitamin A is related to the occurrence of blindness. The photosensitive function of rod cells depends on the visual pigment composed of a molecule of 11-cis retinal and a molecule of opsin and meanwhile vitamin A is the starting material for the synthesis of 11-cis-retinal (Harrison, 2019). Because of the importance of the liver for the transportation and storage of vitamin A, damage to liver function can also lead to vitamin A deficiency in the body. For example, patients undergoing liver transplantation have a high probability of developing vitamin A deficiency (Venu et al., 2013). Therefore, night blindness and other visual disorders are more common in patients with liver disease.

More importantly, it is mostly believed that inflammatory liver disease can easily lead to some eye diseases, and Chinese medicine prescriptions for treating eye diseases have been proven to improve liver and ocular inflammation at the same time, such as lycium barbarum and chrysanthemum. Lycium barbarum polysaccharide(LBP) is the main active ingredient of lycium barbarum (Amagase et al., 2009). Studies have found that LBP is a promising neuron protective agent, which can effectively improve oxidative stress, inflammation, apoptosis and cell death (Xing et al., 2016; Zhong et al., 2020), consequently it can directly and indirectly protect the optic nerve. In addition, LBP also protect the liver. LBP significantly improve the damage induced by non-alcoholic steatohepatitis, including the increase in serum ALT and AST levels, liver oxidative stress, fibrosis, inflammation, and apoptosis (Xiao et al., 2014; Xiao et al., 2018). Chrysanthemum contains luteolin. Studies have found that luteolin has the effect of anti-inflammatory and blocking the production of reactive oxygen species, and has anti-uveitis (Kanai et al., 2016) and anti-retinal neovascularization (Park et al., 2012) effect. In the liver, luteolin and luteolin-7-O-glucoside prevent GalN/LPS-induced hepatotoxicity in mice by regulating inflammatory mediators and antioxidant enzyme activities (Park and Song, 2019), thus play a role in protecting liver. In all, the practice of traditional Chinese medicine provides an integrative view and a novel insight into the underlying correlation between liver and eye. As liver performs a number of essential functions related to detoxication, nutrient storage, metabolism and etc. for the whole system including eye, understanding the liver-eye communicating mechanism, especially the molecular mediators are of great significance.

### Artificial Intelligence Application of Liver-Eye Relationship

With the emergence of graphics processing unit, the progress of mathematical models, the availability of big data and the advent of low-cost sensors, AI has been used in many industries, including the Web of things, social media and medical fields (LeCun et al., 2015). In the medical field, especially in the image-centered departments such as radiology, dermatology, pathology and ophthalmology, AI’s deep learning (DL) techniques have been widely used and made great progress due to their strong graphics processing ability (Schmidt-Erfurth et al., 2018; Ting et al., 2019a). DL approaches used complete images, and associated the entire image with a diagnostic output, thereby eliminating the use of “hand-engineered” image features (Ting et al., 2019b). In ophthalmology, DL system was mainly used in two fields. First, the DL system has been shown to accurately detect DR (Abràmoff et al., 2016; Gargeya and Leng, 2017; Ting et al., 2017), glaucoma (Ting et al., 2017), AMD (Ting et al., 2017; Burlina et al., 2018a; Burlina et al., 2018b), ROP (Brown et al., 2018) and ametropia (Poplin et al., 2018) using fundus image. Secondly, new studies have shown that several retinal conditions, such as CNV, early AMD and diabetic macular edema, can also be accurately detected by the DL algorithm used in optical coherence tomography images (Lee et al., 2017; Ting et al., 2019b). Thereby, AI’s graphics processing ability and DL system make the eye a window for observing systemic diseases as well, and have the advantages of non-invasive examination and diagnosis. For example, using eye manifestations to predict neurodegenerative diseases, diabetes, etc.

Due to its non-invasiveness and convenience, analyzing ocular images via AI has a preliminary advantage in identifying liver diseases, which can also confirm the link between liver and eye from clinical perspective. In the process of liver metabolism, the direct toxicity of abnormal metabolites, excessive normal metabolites and insufficient liver energy metabolism can lead to abnormal ocular performance (Poll-The et al., 2003). Many hepatobiliary diseases, including hepatitis, cirrhosis, HCC and cholelithiasis, are often accompanied by non-specific ocular abnormalities, such as scleral jaundice caused by the accumulation of bilirubin in sclera. In addition, there are also ocular abnormalities in some rare liver diseases, including corneal Kayser-Fleischer ring in Wilson disease, cherry-red macular spots in Niemann-Pick disease, and posterior embryotoxon or optic disc drusen in Alagille syndrome. Therefore, ophthalmological examination is helpful to screen some specific hepatobiliary diseases. In view of this, the AI algorithm for screening liver diseases using ocular models has also been applied and reported for the first time (Xiao et al., 2021). In this AI algorithm, the DL system utilized patient information, including slit-lamp anterior segment photos, fundus photos and diagnostic data of hepatic diseases, to train and adjust to form a reliable DL model. By analyzing the ocular images (anterior segment photos and fundus photos), this model successfully predicted the category of hepatobiliary diseases, including HCC, hepatic cirrhosis, chronic viral hepatitis, NAFLD and cholelithiasis. Additionally, when analyzing the effect-region of the eye images, it is found that the analysis area of the DL model was mainly concentrated in the sclera, iris and the distribution area of the optic disc and inferior vascular arch (Xiao et al., 2021). This indicated that the influences of hepatic diseases on the eyes may be concentrated in these areas as well. However, at current stage, the DL model cannot describe the pathological characteristics of these areas in detail, which is need to be further investigated.

## Future Prospective

In terms of communication between the liver and the eyes, there are still many worthwhile problems that still need to be resolved.

Regarding the molecular aspect, most of the current researches only describe some phenotypic links between the liver and the eye, but do not go into the specific molecular mechanisms for research. For example, how these molecules that embody liver-eye communication are produced, and the induction mechanisms of transcriptional reprogramming, protein translation, modification, and secretion of these molecules are still not well understood. We have found that Notch signaling regulates HGF and angiopoietin secreted by hepatic sinusoidal endothelium, and promotes liver regeneration and fibrosis, suggesting that Notch signaling may further affect ocular diseases by regulating hepatic sinusoidal endothelial cells (Duan et al., 2018). In addition, how these molecules are transported between organs for communication, and the transport mechanism remains to be studied. What’s more, how these molecules are regulated in time and space in the pathological process, and their influence on the occurrence, development and outcome of the disease is still relatively shallow.

At present, the metabolic relationship between liver and eye is mainly reflected in the metabolism of glucose and lipids. They mainly regulate vascular endothelial cells and affect angiogenesis to reflect the liver-eye connection. In particular, the relationship between NAFLD and DR reflects the liver-eye connection. However, the current research on the deep pathogenesis of NAFLD and DR is not clear. There are also some controversies about the correlation between NAFLD and DR. Therefore, more researchers are needed to clarify the correlation between NAFLD and diabetic retinopathy. And underlying molecular mechanisms. In addition, many complications of liver disease in the eye reflect the liver-eye connection. For example, abnormal liver copper metabolism leads to the deposition of copper ions in the eye. However, why is there such a phenomenon that abnormal metabolism of liver disease tends to deposit in the eyes?

Finally, there are some evidences of liver-eye connection in TCM, but in the past, there was no way to explain the mechanism well due to the limitation of medical level. With the development of modern medicine, some of the previously unexplainable problems have also been explained. Therefore, paying attention to the liver-eye connection embodied in TCM may provide researchers with new ideas. Moreover, artificial intelligence is now rapidly developing. Therefore, the use of artificial intelligence to examine the eyes may also provide a new idea for our non-invasive and rapid screening of liver diseases through the connection between the liver and the eye.

In the future, more research on the relationship between liver and eye will help us understand the communication mechanism between liver and eye more clearly, which will help us better understand the pathogenesis and progression of these liver diseases or eye diseases, and more contribute to clinical treatment and the development of new therapeutic targets. Moreover, the research on the communication between the liver and the eye will inspire more organs and the mechanism of communication between organs, and it helps us to understand more clearly how the human body, a sophisticated and complex system can conduct steady-state regulation, which is conducive to revealing the mysteries of the human body.

## References

[B1] Abdelmoemen G. Khodeir S. A. Zaki A. N. Kassab M. Abou-Saif S. Abd-Elsalam S. (2019). Overexpression of Hepassocin in Diabetic Patients with Nonalcoholic Fatty Liver Disease May Facilitate Increased Hepatic Lipid Accumulation. Endocr. Metab. Immune Disord. Drug Targets 19 (2), 185–188. 10.2174/1871530318666180716100543 30009716

[B2] Abràmoff M. D. Lou Y. Erginay A. Clarida W. Amelon R. Folk J. C. (2016). Improved Automated Detection of Diabetic Retinopathy on a Publicly Available Dataset through Integration of Deep Learning. Invest. Ophthalmol. Vis. Sci. 57 (13), 5200–5206. 10.1167/iovs.16-19964 27701631

[B3] Abu-Farha M. Ghosh A. Al-Khairi I. Madiraju S. R. M. Abubaker J. Prentki M. (2020). The Multi-Faces of Angptl8 in Health and Disease: Novel Functions beyond Lipoprotein Lipase Modulation. Prog. Lipid Res. 80, 101067. 10.1016/j.plipres.2020.101067 33011191

[B4] Adams L. A. Waters O. R. Knuiman M. W. Elliott R. R. Olynyk J. K. (2009). NAFLD as a Risk Factor for the Development of Diabetes and the Metabolic Syndrome: an Eleven-Year Follow-Up Study. Am. J. Gastroenterol. 104 (4), 861–867. 10.1038/ajg.2009.67 19293782

[B5] Afarideh M. Aryan Z. Ghajar A. Ganji M. Ghaemi F. Saadat M. (2019). Association of Non-alcoholic Fatty Liver Disease with Microvascular Complications of Type 2 Diabetes. Prim. Care Diabetes 13 (6), 505–514. 10.1016/j.pcd.2019.03.009 31054837

[B6] Amagase H. Sun B. Borek C. (2009). Lycium Barbarum (goji) Juice Improves *In Vivo* Antioxidant Biomarkers in Serum of Healthy Adults. Nutr. Res. 29 (1), 19–25. 10.1016/j.nutres.2008.11.005 19185773

[B7] Ammar T. Auwzinger G. Michaelides M. Michaelides M. (2003). Cortical Blindness and Hepatic Encephalopathy. Acta Ophthalmol. Scand. 81 (4), 402–404. 10.1034/j.1600-0420.2003.00093.x 12859270

[B8] Azuma S. Asahina Y. Kakinuma S. Azuma K. Miyoshi M. Inoue E. (2019). Diabetic Retinopathy as a Risk Factor Associated with the Development of Hepatocellular Carcinoma in Nonalcoholic Fatty Liver Disease. Dig. Dis. 37 (3), 247–254. 10.1159/000493580 30625487

[B9] Baumann B. H. Shu W. Song Y. Sterling J. Kozmik Z. Lakhal-Littleton S. (2019). Liver-Specific, but Not Retina-specific, Hepcidin Knockout Causes Retinal Iron Accumulation and Degeneration. Am. J. Pathol. 189 (9), 1814–1830. 10.1016/j.ajpath.2019.05.022 31287995 PMC6723216

[B10] Brown J. M. Campbell J. P. Beers A. Chang K. Ostmo S. Chan R. V. P. (2018). Automated Diagnosis of Plus Disease in Retinopathy of Prematurity Using Deep Convolutional Neural Networks. JAMA Ophthalmol. 136 (7), 803–810. 10.1001/jamaophthalmol.2018.1934 29801159 PMC6136045

[B11] Burlina P. Joshi N. Pacheco K. D. Freund D. E. Kong J. Bressler N. M. (2018). Utility of Deep Learning Methods for Referability Classification of Age-Related Macular Degeneration. JAMA Ophthalmol. 136 (11), 1305–1307. 10.1001/jamaophthalmol.2018.3799 30193354 PMC6248178

[B12] Burlina P. M. Joshi N. Pacheco K. D. Freund D. E. Kong J. Bressler N. M. (2018). Use of Deep Learning for Detailed Severity Characterization and Estimation of 5-Year Risk Among Patients with Age-Related Macular Degeneration. JAMA Ophthalmol. 136 (12), 1359–1366. 10.1001/jamaophthalmol.2018.4118 30242349 PMC6583826

[B13] Campos E. J. Campos A. Martins J. Ambrósio A. F. (2017). Opening Eyes to Nanomedicine: Where We Are, Challenges and Expectations on Nanotherapy for Diabetic Retinopathy. Nanomedicine: Nanotechnology, Biol. Med. 13 (6), 2101–2113. 10.1016/j.nano.2017.04.008 28428052

[B14] Carroll W. J. Peck T. Jenkins T. L. Karcioglu Z. A. (2017). Periocular, Periorbital, and Orbital Pathology in Liver Disease. Surv. Ophthalmol. 62 (2), 134–149. 10.1016/j.survophthal.2016.11.002 27863968

[B15] Chen E.-B. Zhou S.-L. Pang X.-G. Yin D. Miao P.-Z. Yang Y. (2017). Prostate-derived ETS Factor Improves Prognosis and Represses Proliferation and Invasion in Hepatocellular Carcinoma. Oncotarget 8 (32), 52488–52500. 10.18632/oncotarget.14924 28881746 PMC5581045

[B16] Chen L. Wang K. Long A. Jia L. Zhang Y. Deng H. (2017). Fasting-induced Hormonal Regulation of Lysosomal Function. Cell Res 27 (6), 748–763. 10.1038/cr.2017.45 28374748 PMC5518872

[B17] Cipriani V. Lorés-Motta L. He F. Fathalla D. Tilakaratna V. McHarg S. (2020). Increased Circulating Levels of Factor H-Related Protein 4 are Strongly Associated with Age-Related Macular Degeneration. Nat. Commun. 11 (1), 778. 10.1038/s41467-020-14499-3 32034129 PMC7005798

[B18] Clemons T. E. Milton R. C. Klein R. Seddon J. M. Ferris F. L. (2005). Risk Factors for the Incidence of Advanced Age-Related Macular Degeneration in the Age-Related Eye Disease Study (AREDS) AREDS Report No. 19. Ophthalmology 112 (4), 533–539. 10.1016/j.ophtha.2004.10.047 15808240 PMC1513667

[B19] D L. Wp G. (2021). Clinical Study of TCM Syndrome Differentiation in the Treatment of Dry Eye. Chin. J. Ophthalmol. 31 (05), 331–336.

[B20] Duan J.-L. Ruan B. Yan X.-C. Liang L. Song P. Yang Z.-Y. (2018). Endothelial Notch Activation Reshapes the Angiocrine of Sinusoidal Endothelia to Aggravate Liver Fibrosis and blunt Regeneration in Mice. Hepatology 68 (2), 677–690. 10.1002/hep.29834 29420858 PMC6099357

[B21] Elahy M. Baindur-Hudson S. Cruzat V. F. Newsholme P. Dass C. R. (2014). Mechanisms of PEDF-Mediated protection against Reactive Oxygen Species Damage in Diabetic Retinopathy and Neuropathy. J. Endocrinol. 222 (3), R129–R139. 10.1530/JOE-14-0065 24928938

[B22] Fahnehjelm K. T. Fischler B. Martin L. Nemeth A. (2011). Occurrence and Pattern of Ocular Disease in Children with Cholestatic Disorders. Acta ophthalmologica 89 (2), 143–150. 10.1111/j.1755-3768.2009.01671.x 20384607

[B23] Farrell G. C. Larter C. Z. (2006). Nonalcoholic Fatty Liver Disease: from Steatosis to Cirrhosis. Hepatology 43 (2 Suppl. 1), S99–S112. 10.1002/hep.20973 16447287

[B24] Fleming C. R. Dickson E. R. Wahner H. W. Hollenhorst R. W. McCall J. T. (1977). Pigmented Corneal Rings in Non-wilsonian Liver Disease. Ann. Intern. Med. 86 (3), 285–288. 10.7326/0003-4819-86-3-285 842986

[B25] Frenzel C. Teschke R. (2016). Herbal Hepatotoxicity: Clinical Characteristics and Listing Compilation. Int. J. Mol. Sci. 17 (5), 588. 10.3390/ijms17050588 PMC4881436 27128912

[B26] Fu Z. Gong Y. Liegl R. Wang Z. Liu C.-H. Meng S. S. (2017). FGF21 Administration Suppresses Retinal and Choroidal Neovascularization in Mice. Cell Rep. 18 (7), 1606–1613. 10.1016/j.celrep.2017.01.014 28199833 PMC5328201

[B27] Fu Z. Wang Z. Liu C.-H. Gong Y. Cakir B. Liegl R. (2018). Fibroblast Growth Factor 21 Protects Photoreceptor Function in Type 1 Diabetic Mice. Diabetes 67 (5), 974–985. 10.2337/db17-0830 29487115 PMC5909994

[B28] Fujihara M. Bartels E. Nielsen L. B. Handa J. T. (2009). A Human apoB100 Transgenic Mouse Expresses Human apoB100 in the RPE and Develops Features of Early AMD. Exp. Eye Res. 88 (6), 1115–1123. 10.1016/j.exer.2009.01.017 19450445 PMC2729121

[B29] Gaich G. Chien J. Y. Fu H. Glass L. C. Deeg M. A. Holland W. L. (2013). The Effects of LY2405319, an FGF21 Analog, in Obese Human Subjects with Type 2 Diabetes. Cel Metab. 18 (3), 333–340. 10.1016/j.cmet.2013.08.005 24011069

[B30] Gardner T. W. Abcouwer S. F. Barber A. J. Jackson G. R. (2011). An Integrated Approach to Diabetic Retinopathy Research. Arch. Ophthalmol. 129 (2), 230–235. 10.1001/archophthalmol.2010.362 21320973 PMC3086099

[B31] Gargeya R. Leng T. (2017). Automated Identification of Diabetic Retinopathy Using Deep Learning. Ophthalmology 124 (7), 962–969. 10.1016/j.ophtha.2017.02.008 28359545

[B32] Geng L. Lam K. S. L. Xu A. (2020). The Therapeutic Potential of FGF21 in Metabolic Diseases: from Bench to Clinic. Nat. Rev. Endocrinol. 16 (11), 654–667. 10.1038/s41574-020-0386-0 32764725

[B33] Golestaneh N. Chu Y. Xiao Y.-Y. Stoleru G. L. Theos A. C. (2017). Dysfunctional Autophagy in RPE, a Contributing Factor in Age-Related Macular Degeneration. Cell Death Dis 8 (1), e2537. 10.1038/cddis.2016.453 28055007 PMC5386365

[B34] Grierson I. Heathcote L. Hiscott P. Hogg P. Briggs M. Hagan S. (2000). Hepatocyte Growth Factor/scatter Factor in the Eye. Prog. Retin. Eye Res. 19 (6), 779–802. 10.1016/s1350-9462(00)00015-x 11029554

[B35] Harrison E. H. (2019). Mechanisms of Transport and Delivery of Vitamin A and Carotenoids to the Retinal Pigment Epithelium. Mol. Nutr. Food Res. 63 (15), 1801046. 10.1002/mnfr.201801046 30698921

[B36] Hazlehurst J. M. Woods C. Marjot T. Cobbold J. F. Tomlinson J. W. (2016). Non-alcoholic Fatty Liver Disease and Diabetes. Metabolism 65 (8), 1096–1108. 10.1016/j.metabol.2016.01.001 26856933 PMC4943559

[B37] He L. Deng L. Zhang Q. Guo J. Zhou J. Song W. (2017). Diagnostic Value of CK-18, FGF-21, and Related Biomarker Panel in Nonalcoholic Fatty Liver Disease: A Systematic Review and Meta-Analysis. Biomed. Res. Int. 2017, 1–12. 10.1155/2017/9729107 PMC5343245 28326329

[B38] Heesterbeek T. J. Lorés‐Motta L. Hoyng C. B. Lechanteur Y. T. E. den Hollander A. I. (2020). Risk Factors for Progression of Age‐related Macular Degeneration. Ophthalmic Physiol. Opt. 40 (2), 140–170. 10.1111/opo.12675 32100327 PMC7155063

[B39] Hs B. Zn Y. Qx Z. Xf X. Xb H. Jk S. (2015). Discussion on TCM Syndrome Differentiation Theory and Treatment of Uveitis. J. Shandong Traditional Chin. Med. Univ. 39 (01), 34–36.

[B40] Hughes A. E. Bridgett S. Meng W. Li M. Curcio C. A. Stambolian D. (2016). Sequence and Expression of Complement Factor H Gene Cluster Variants and Their Roles in Age-Related Macular Degeneration Risk. Invest. Ophthalmol. Vis. Sci. 57 (6), 2763–2769. 10.1167/iovs.15-18744 27196323 PMC4884056

[B41] J P. Ys Z. Kz C. Y W. P L. Jq L. (2017). Professor Peng Qinghua Used Xiaoyao Powder to Treat Ophthalmic Diseases. J. Hunan Traditional Chin. Med. Univ. 37 (1), 45–47.

[B42] Jun S. Datta S. Wang L. Pegany R. Cano M. Handa J. T. (2019). The Impact of Lipids, Lipid Oxidation, and Inflammation on AMD, and the Potential Role of miRNAs on Lipid Metabolism in the RPE. Exp. Eye Res. 181, 346–355. 10.1016/j.exer.2018.09.023 30292489 PMC6443454

[B43] Kanai K. Nagata S. Hatta T. Sugiura Y. Sato K. Yamashita Y. (2016). Therapeutic Anti-inflammatory Effects of Luteolin on Endotoxin-Induced Uveitis in Lewis Rats. J. Vet. Med. Sci. 78 (8), 1381–1384. 10.1292/jvms.16-0196 27170432 PMC5053947

[B44] Keuper M. Häring H.-U. Staiger H. (2020). Circulating FGF21 Levels in Human Health and Metabolic Disease. Exp. Clin. Endocrinol. Diabetes 128 (11), 752–770. 10.1055/a-0879-2968 31108554

[B45] Kim B.-Y. Jung C.-H. Mok J.-O. Kang S. K. Kim C.-H. (2014). Prevalences of Diabetic Retinopathy and Nephropathy Are Lower in Korean Type 2 Diabetic Patients with Non-alcoholic Fatty Liver Disease. J. Diabetes Invest. 5 (2), 170–175. 10.1111/jdi.12139 PMC4023580 24843757

[B46] Kim J. E. Clark R. M. Park Y. Lee J. Fernandez M. L. (2012). Lutein Decreases Oxidative Stress and Inflammation in Liver and Eyes of guinea Pigs Fed a Hypercholesterolemic Diet. Nutr. Res. Pract. 6 (2), 113–119. 10.4162/nrp.2012.6.2.113 22586499 PMC3349032

[B47] Klein R. J. Zeiss C. Chew E. Y. Tsai J.-Y. Sackler R. S. Haynes C. (2005). Complement Factor H Polymorphism in Age-Related Macular Degeneration. Science 308 (5720), 385–389. 10.1126/science.1109557 15761122 PMC1512523

[B48] Lazo M. Clark J. (2008). The Epidemiology of Nonalcoholic Fatty Liver Disease: a Global Perspective. Semin. Liver Dis. 28 (4), 339–350. 10.1055/s-0028-1091978 18956290

[B49] LeCun Y. Bengio Y. Hinton G. (2015). Deep Learning. Nature 521 (7553), 436–444. 10.1038/nature14539 26017442

[B50] Lee C. S. Tyring A. J. Deruyter N. P. Wu Y. Rokem A. Lee A. Y. (2017). Deep-learning Based, Automated Segmentation of Macular Edema in Optical Coherence Tomography. Biomed. Opt. Express 8 (7), 3440–3448. 10.1364/BOE.8.003440 28717579 PMC5508840

[B51] Lee D. Tomita Y. Jeong H. Miwa Y. Tsubota K. Negishi K. (2021). Pemafibrate Prevents Retinal Dysfunction in a Mouse Model of Unilateral Common Carotid Artery Occlusion. Int. J. Mol. Sci. 22 (17), 9408. 10.3390/ijms22179408 34502311 PMC8431531

[B52] Leite N. C. Cardoso C. R. L. Salles G. F. (2021). Importance of Non-invasive Liver Fibrosis Scores for Mortality and Complications Development in Individuals with Type 2 Diabetes. J. Diabetes Complications 35 (5), 107879. 10.1016/j.jdiacomp.2021.107879 33573891

[B53] Li L. H. Lee J. C.-Y. Leung H. H. Lam W. C. Fu Z. Lo A. C. Y. (2020). Lutein Supplementation for Eye Diseases. Nutrients 12 (6), 1721. 10.3390/nu12061721 PMC7352796 32526861

[B54] Li X. Sun X. Carmeliet P. (2019). Hallmarks of Endothelial Cell Metabolism in Health and Disease. Cel Metab. 30 (3), 414–433. 10.1016/j.cmet.2019.08.011 31484054

[B55] Lin T.-Y. Chen Y.-J. Chen W.-L. Peng T.-C. (2016). The Relationship between Nonalcoholic Fatty Liver Disease and Retinopathy in NHANES III. PLoS One 11 (11), e0165970. 10.1371/journal.pone.0165970 27802330 PMC5089732

[B56] Lin Y. Xiao Y.-c. Zhu H. Xu Q.-y. Qi L. Wang Y.-b. (2014). Serum Fibroblast Growth Factor 21 Levels Are Correlated with the Severity of Diabetic Retinopathy. J. Diabetes Res. 2014, 1–6. 10.1155/2014/929756 PMC4009259 24895642

[B57] Lin Z. Tian H. Lam K. S. L. Lin S. Hoo R. C. L. Konishi M. (2013). Adiponectin Mediates the Metabolic Effects of FGF21 on Glucose Homeostasis and Insulin Sensitivity in Mice. Cell Metab. 17 (5), 779–789. 10.1016/j.cmet.2013.04.005 23663741

[B58] Liu Y. Zheng Y. Zhou Y. Liu Y. Xie M. Meng W. (2020). The Expression and Significance of mTORC1 in Diabetic Retinopathy. BMC Ophthalmol. 20 (1), 297. 10.1186/s12886-020-01553-3 32689970 PMC7370483

[B59] Lu Q. Lu L. Chen W. Lu P. (2017). Expression of Angiopoietin-like Protein 8 Correlates with VEGF in Patients with Proliferative Diabetic Retinopathy. Graefes Arch. Clin. Exp. Ophthalmol. 255 (8), 1515–1523. 10.1007/s00417-017-3676-z 28456825

[B60] Lu Q. Lu P. Chen W. Lu L. Zheng Z. (2018). ANGPTL-4 Induces Diabetic Retinal Inflammation by Activating Profilin-1. Exp. Eye Res. 166, 140–150. 10.1016/j.exer.2017.10.009 29031854

[B61] Lu Q. Zou W. Chen B. Zou C. Zhao M. Zheng Z. (2016). ANGPTL-4 Correlates with Vascular Endothelial Growth Factor in Patients with Proliferative Diabetic Retinopathy. Graefes Arch. Clin. Exp. Ophthalmol. 254 (7), 1281–1288. 10.1007/s00417-015-3187-8 26483143

[B62] Lundåsen T. Hunt M. C. Nilsson L. M. Sanyal S. Angelin B. Alexson S. E. (2007). PPARalpha Is a Key Regulator of Hepatic FGF21. Biochem. Biophys. Res. Commun. 360 (2), 437–440. 10.1016/j.bbrc.2007.06.068 17601491

[B63] Lv W.-S. Sun R. X. Gao Y. Y. Wen J. P. Pan R. F. Li L. (2013). Nonalcoholic Fatty Liver Disease and Microvascular Complications in Type 2 Diabetes. World J. Gastroenterol. 19 (20), 3134–3142. 10.3748/wjg.v19.i20.3134 23716995 PMC3662955

[B64] Lyzogubov V. V. Tytarenko R. G. Jha P. Liu J. Bora N. S. Bora P. S. (2010). Role of Ocular Complement Factor H in a Murine Model of Choroidal Neovascularization. Am. J. Pathol. 177 (4), 1870–1880. 10.2353/ajpath.2010.091168 20813971 PMC2947282

[B65] Malek G. Johnson L. V. Mace B. E. Saloupis P. Schmechel D. E. Rickman D. W. (2005). Apolipoprotein E Allele-dependent Pathogenesis: A Model for Age-Related Retinal Degeneration. Proc. Natl. Acad. Sci. 102 (33), 11900–11905. 10.1073/pnas.0503015102 16079201 PMC1187976

[B66] Mandal N. A. Ayyagari R. (2006). Complement Factor H: Spatial and Temporal Expression and Localization in the Eye. Invest. Ophthalmol. Vis. Sci. 47 (9), 4091–4097. 10.1167/iovs.05-1655 16936129

[B67] Mao Y. Zhu L. Huang Z. Luo C. Zhou T. Li L. (2020). Stem-like Tumor Cells Involved in Heterogeneous Vasculogenesis in Breast Cancer. Endocr. Relat. Cancer 27 (1), 23–39. 10.1530/ERC-19-0054 31705798

[B68] Markan K. R. Naber M. C. Ameka M. K. Anderegg M. D. Mangelsdorf D. J. Kliewer S. A. (2014). Circulating FGF21 Is Liver Derived and Enhances Glucose Uptake during Refeeding and Overfeeding. Diabetes 63 (12), 4057–4063. 10.2337/db14-0595 25008183 PMC4238010

[B69] Matsumoto K. Ishikawa H. Nishimura D. Hamasaki K. Nakao K. Eguchi K. (2004). Antiangiogenic Property of Pigment Epithelium-Derived Factor in Hepatocellular Carcinoma. Hepatology 40 (1), 252–259. 10.1002/hep.20259 15239109

[B70] Meex R. C. R. Watt M. J. (2017). Hepatokines: Linking Nonalcoholic Fatty Liver Disease and Insulin Resistance. Nat. Rev. Endocrinol. 13 (9), 509–520. 10.1038/nrendo.2017.56 28621339

[B71] Mejias M. Coch L. Berzigotti A. Garcia-Pras E. Gallego J. Bosch J. (2015). Antiangiogenic and Antifibrogenic Activity of Pigment Epithelium-Derived Factor (PEDF) in Bile Duct-Ligated portal Hypertensive Rats. Gut 64 (4), 657–666. 10.1136/gutjnl-2014-307138 24848263

[B72] Mertz J. L. Sripathi S. R. Yang X. Chen L. Esumi N. Zhang H. (2021). Proteomic and Phosphoproteomic Analyses Identify Liver-Related Signaling in Retinal Pigment Epithelial Cells during EMT. Cell Rep. 37 (3), 109866. 10.1016/j.celrep.2021.109866 34686321

[B73] Mikolasevic I. Rahelic D. Turk-Wensween T. Ruzic A. Domislovic V. Hauser G. (2021). Significant Liver Fibrosis, as Assessed by Fibroscan, Is Independently Associated with Chronic Vascular Complications of Type 2 Diabetes: A Multicenter Study. Diabetes Res. Clin. Pract. 177, 108884. 10.1016/j.diabres.2021.108884 34082054

[B74] Mima A. (2016). Incretin-Based Therapy for Prevention of Diabetic Vascular Complications. J. Diabetes Res. 2016, 1–12. 10.1155/2016/1379274 PMC4735992 26881236

[B75] Morovat A. Weerasinghe G. Nesbitt V. Hofer M. Agnew T. Quaghebeur G. (2017). Use of FGF-21 as a Biomarker of Mitochondrial Disease in Clinical Practice. J. Clin. Med. 6 (8), 80. 10.3390/jcm6080080 PMC5575582 28825656

[B76] Nakabayashi M. Morishita R. Nakagami H. Kuba K. Matsumoto K. Nakamura T. (2003). HGF/NK4 Inhibited VEGF-Induced Angiogenesis in *In Vitro* Cultured Endothelial Cells and *In Vivo* Rabbit Model. Diabetologia 46 (1), 115–123. 10.1007/s00125-002-0954-y 12637990

[B77] Nguyen P. Leray V. Diez M. Serisier S. Bloc’h J. L. Siliart B. (2008). Liver Lipid Metabolism. J. Anim. Physiol. Anim. Nutr. 92 (3), 272–283. 10.1111/j.1439-0396.2007.00752.x 18477307

[B78] Park C. M. Song Y.-S. (2019). Luteolin and Luteolin-7-O-Glucoside Protect against Acute Liver Injury through Regulation of Inflammatory Mediators and Antioxidative Enzymes in GalN/LPS-Induced Hepatitic ICR Mice. Nutr. Res. Pract. 13 (6), 473–479. 10.4162/nrp.2019.13.6.473 31814922 PMC6883227

[B79] Park S. W. Cho C. S. Jun H. O. Ryu N. H. Kim J. H. Yu Y. S. (2012). Anti-angiogenic Effect of Luteolin on Retinal Neovascularization via Blockade of Reactive Oxygen Species Production. Invest. Ophthalmol. Vis. Sci. 53 (12), 7718–7726. 10.1167/iovs.11-8790 23099493

[B80] Perdices L. Fuentes-Broto L. Segura F. Ben Gdara N. Sánchez-Cano A. I. Insa G. (2018). Hepatic Oxidative Stress in Pigmented P23H Rhodopsin Transgenic Rats with Progressive Retinal Degeneration. Free Radic. Biol. Med. 124, 550–557. 10.1016/j.freeradbiomed.2018.07.005 30006118

[B81] Perumpail B. J. Khan M. A. Yoo E. R. Cholankeril G. Kim D. Ahmed A. (2017). Clinical Epidemiology and Disease burden of Nonalcoholic Fatty Liver Disease. World J. Gastroenterol. 23 (47), 8263–8276. 10.3748/wjg.v23.i47.8263 29307986 PMC5743497

[B82] Poll-The B. T. Maillette de Buy Wenniger-Prick L. J. Barth P. G. Duran M. (2003). The Eye as a Window to Inborn Errors of Metabolism. J. Inherit. Metab. Dis. 26 (2–3), 229–244. 10.1023/a:1024493318913 12889663

[B83] Poplin R. Varadarajan A. V. Blumer K. Liu Y. McConnell M. V. Corrado G. S. (2018). Prediction of Cardiovascular Risk Factors from Retinal Fundus Photographs via Deep Learning. Nat. Biomed. Eng. 2 (3), 158–164. 10.1038/s41551-018-0195-0 31015713

[B84] Potthoff M. J. Inagaki T. Satapati S. Ding X. He T. Goetz R. (2009). FGF21 Induces PGC-1 and Regulates Carbohydrate and Fatty Acid Metabolism during the Adaptive Starvation Response. Proc. Natl. Acad. Sci. 106 (26), 10853–10858. 10.1073/pnas.0904187106 19541642 PMC2705613

[B85] Potthoff M. J. Kliewer S. A. Mangelsdorf D. J. (2012). Endocrine Fibroblast Growth Factors 15/19 and 21: from Feast to Famine. Genes Dev. 26 (4), 312–324. 10.1101/gad.184788.111 22302876 PMC3289879

[B86] Protiva P. Gong J. Sreekumar B. Torres R. Zhang X. Belinsky G. S. (2015). Pigment Epithelium-Derived Factor (PEDF) Inhibits Wnt/β-Catenin Signaling in the Liver. Cell Mol. Gastroenterol. Hepatol. 1 (5), 535–549. 10.1016/j.jcmgh.2015.06.006 26473164 PMC4604042

[B87] Richard J. M. Friendly D. S. (1983). Ocular Findings in Pediatric Systemic Disease. Pediatr. Clin. North America 30 (6), 1123–1144. 10.1016/s0031-3955(16)34506-0 6316240

[B88] Roden M. Shulman G. I. (2019). The Integrative Biology of Type 2 Diabetes. Nature 576 (7785), 51–60. 10.1038/s41586-019-1797-8 31802013

[B89] Romeo S. Yin W. Kozlitina J. Pennacchio L. A. Boerwinkle E. Hobbs H. H. (2009). Rare Loss-Of-Function Mutations in ANGPTL Family Members Contribute to Plasma Triglyceride Levels in Humans. J. Clin. Invest. 119 (1), 70–79. 10.1172/JCI37118 19075393 PMC2613476

[B90] Schmidt-Erfurth U. Bogunovic H. Sadeghipour A. Schlegl T. Langs G. Gerendas B. S. (2018). Machine Learning to Analyze the Prognostic Value of Current Imaging Biomarkers in Neovascular Age-Related Macular Degeneration. Ophthalmol. Retina 2 (1), 24–30. 10.1016/j.oret.2017.03.015 31047298

[B91] Schoors S. Bruning U. Missiaen R. Queiroz K. C. S. Borgers G. Elia I. (2015). Fatty Acid Carbon Is Essential for dNTP Synthesis in Endothelial Cells. Nature 520 (7546), 192–197. 10.1038/nature14362 25830893 PMC4413024

[B92] Shields J. A. Shields C. L. Donoso L. A. (1991). Management of Posterior Uveal Melanoma. Surv. Ophthalmol. 36 (3), 161–195. 10.1016/0039-6257(91)90001-v 1776122

[B93] Song D. Li C. Wang Z. Zhao Y. Shen B. Zhao W. (2021). Association of Non‐alcoholic Fatty Liver Disease with Diabetic Retinopathy in Type 2 Diabetic Patients: A Meta‐analysis of Observational Studies. J. Diabetes Investig. 12 (8), 1471–1479. 10.1111/jdi.13489 PMC8354494 33372390

[B94] Sun X. Lv Y. Huang L. Gao H. Ren C. Li J. (2020). Pro-inflammatory Cytokines Serve as Communicating Molecules between the Liver and Brain for Hepatic Encephalopathy Pathogenesis and Lycium Barbarum Polysaccharides protection. J. Ethnopharmacology 248, 112357. 10.1016/j.jep.2019.112357 31693919

[B95] Targher G. Bertolini L. Rodella S. Zoppini G. Lippi G. Day C. (2008). Non-alcoholic Fatty Liver Disease Is Independently Associated with an Increased Prevalence of Chronic Kidney Disease and Proliferative/laser-Treated Retinopathy in Type 2 Diabetic Patients. Diabetologia 51 (3), 444–450. 10.1007/s00125-007-0897-4 18058083

[B96] Ting D. S. W. Cheung C. Y.-L. Lim G. Tan G. S. W. Quang N. D. Gan A. (2017). Development and Validation of a Deep Learning System for Diabetic Retinopathy and Related Eye Diseases Using Retinal Images from Multiethnic Populations with Diabetes. JAMA 318 (22), 2211–2223. 10.1001/jama.2017.18152 29234807 PMC5820739

[B97] Ting D. S. W. Pasquale L. R. Peng L. Campbell J. P. Lee A. Y. Raman R. (2019). Artificial Intelligence and Deep Learning in Ophthalmology. Br. J. Ophthalmol. 103 (2), 167–175. 10.1136/bjophthalmol-2018-313173 30361278 PMC6362807

[B98] Ting D. S. W. Peng L. Varadarajan A. V. Keane P. A. Burlina P. M. Chiang M. F. (2019). Deep Learning in Ophthalmology: The Technical and Clinical Considerations. Prog. Retin. Eye Res. 72, 100759. 10.1016/j.preteyeres.2019.04.003 31048019

[B99] Tombran-Tink J. Johnson L. V. (1989). Neuronal Differentiation of Retinoblastoma Cells Induced by Medium Conditioned by Human RPE Cells. Invest. Ophthalmol. Vis. Sci. 30 (8), 1700–1707. 2668219

[B100] Tomita Y. Fu Z. Wang Z. Cakir B. Cho S. S. Britton W. (2020). Long-Acting FGF21 Inhibits Retinal Vascular Leakage in *In Vivo* and *In Vitro* Models. Int. J. Mol. Sci. 21 (4), 1188. 10.3390/ijms21041188 PMC7072824 32054022

[B101] Tomita Y. Lee D. Miwa Y. Jiang X. Ohta M. Tsubota K. (2020). Pemafibrate Protects against Retinal Dysfunction in a Murine Model of Diabetic Retinopathy. Int. J. Mol. Sci. 21 (17), 6243. 10.3390/ijms21176243 PMC7503472 32872333

[B102] Tomita Y. Ozawa N. Miwa Y. Ishida A. Ohta M. Tsubota K. (2019). Pemafibrate Prevents Retinal Pathological Neovascularization by Increasing FGF21 Level in a Murine Oxygen-Induced Retinopathy Model. Int. J. Mol. Sci. 20 (23), 5878. 10.3390/ijms20235878 PMC6928689 31771164

[B103] Toomey C. B. Kelly U. Saban D. R. Bowes Rickman C. (2015). Regulation of Age-Related Macular Degeneration-like Pathology by Complement Factor H. Proc. Natl. Acad. Sci. USA 112 (23), E3040–E3049. 10.1073/pnas.1424391112 25991857 PMC4466717

[B104] Tsygankova P. G. Itkis Y. S. Krylova T. D. Kurkina M. V. Bychkov I. O. Ilyushkina A. A. (2019). Plasma FGF‐21 and GDF‐15 Are Elevated in Different Inherited Metabolic Diseases and Are Not Diagnostic for Mitochondrial Disorders. J. Inher Metab. Disea 42 (5), 918–933. 10.1002/jimd.12142 31260105

[B105] Tucker B. Li H. Long X. Rye K.-A. Ong K. L. (2019). Fibroblast Growth Factor 21 in Non-alcoholic Fatty Liver Disease. Metabolism 101, 153994. 10.1016/j.metabol.2019.153994 31672443

[B106] Unnikrishnan R. Pradeepa R. Joshi S. R. Mohan V. (2017). Type 2 Diabetes: Demystifying the Global Epidemic. Diabetes 66 (6), 1432–1442. 10.2337/db16-0766 28533294

[B107] Vendhan R. Amutha A. Anjana R. M. Unnikrishnan R. Mohan V. (2014). Clinical Profile of Nonalcoholic Fatty Liver Disease Among Young Patients with Type 1 Diabetes Mellitus Seen at a Diabetes Speciality Center in India. Endocr. Pract. 20 (12), 1249–1257. 10.4158/EP14044.OR 25100370

[B108] Venu M. Martin E. Saeian K. Gawrieh S. (2013). High Prevalence of Vitamin A Deficiency and Vitamin D Deficiency in Patients Evaluated for Liver Transplantation. Liver Transpl. 19 (6), 627–633. 10.1002/lt.23646 23495130 PMC3667969

[B109] Walker V. (2014). Ammonia Metabolism and Hyperammonemic Disorders. Adv. Clin. Chem. 67, 73–150. 10.1016/bs.acc.2014.09.002 25735860

[B110] Wang F. So K.-F. Xiao J. Wang H. (2021). Organ-Organ Communication: The Liver’s Perspective. Theranostics 11 (7), 3317–3330. 10.7150/thno.55795 33537089 PMC7847667

[B111] Watt M. J. Miotto P. M. De Nardo W. Montgomery M. K. (2019). The Liver as an Endocrine Organ-Linking NAFLD and Insulin Resistance. Endocr. Rev. 40 (5), 1367–1393. 10.1210/er.2019-00034 31098621

[B112] Wen X. Zhou X. Chen D. Cheng J. Ji L. (2021). Association Between Non-Alcoholic Fatty Liver Disease and Diabetes-Related Microvascular Complications: A Retrospective Cross-Sectional Study of Hospitalized Patients. Endocr. Pract. 2021, S1530-891X(21)00046-X. 10.1016/j.eprac.2021.02.004 33601024

[B113] Wu G. Liu B. Wu Q. Tang C. Du Z. Fang Y. (2021). Correlations between Different Angiogenic and Inflammatory Factors in Vitreous Fluid of Eyes With Proliferative Diabetic Retinopathy. Front. Med. 8, 727407. 10.3389/fmed.2021.727407 PMC8505670 34650995

[B114] Xiao J. Wang F. Liong E. C. So K.-F. Tipoe G. L. (2018). Lycium Barbarum Polysaccharides Improve Hepatic Injury through NFkappa-B and NLRP3/6 Pathways in a Methionine Choline Deficient Diet Steatohepatitis Mouse Model. Int. J. Biol. macromolecules 120 (Pt B), 1480–1489. 10.1016/j.ijbiomac.2018.09.151 30266645

[B115] Xiao J. Xing F. Huo J. Fung M. L. Liong E. C. Ching Y. P. (2014). Lycium Barbarum Polysaccharides Therapeutically Improve Hepatic Functions in Non-alcoholic Steatohepatitis Rats and Cellular Steatosis Model. Sci. Rep. 4, 5587. 10.1038/srep05587 24998389 PMC4083265

[B116] Xiao W. Huang X. Wang J. H. Lin D. R. Zhu Y. Chen C. (2021). Screening and Identifying Hepatobiliary Diseases through Deep Learning Using Ocular Images: a Prospective, Multicentre Study. The Lancet Digital Health 3 (2), e88–e97. 10.1016/s2589-7500(20)30288-0 33509389

[B117] Xing X. Liu F. Xiao J. So K. F. (2016). Neuro-protective Mechanisms of Lycium Barbarum. Neuromol Med. 18 (3), 253–263. 10.1007/s12017-016-8393-y 27033360

[B118] Xu J. Lloyd D. J. Hale C. Stanislaus S. Chen M. Sivits G. (2009). Fibroblast Growth Factor 21 Reverses Hepatic Steatosis, Increases Energy Expenditure, and Improves Insulin Sensitivity in Diet-Induced Obese Mice. Diabetes 58 (1), 250–259. 10.2337/db08-0392 18840786 PMC2606881

[B119] Xu Q. Cao S. Rajapakse S. Matsubara J. A. (2018). Understanding AMD by Analogy: Systematic Review of Lipid-Related Common Pathogenic Mechanisms in AMD, AD, AS and GN. Lipids Health Dis. 17 (1), 3. 10.1186/s12944-017-0647-7 29301530 PMC5755337

[B120] Xu Y. An X. Guo X. Habtetsion T. G. Wang Y. Xu X. (2014). Endothelial PFKFB3 Plays a Critical Role in Angiogenesis. Arterioscler Thromb. Vasc. Biol. 34 (6), 1231–1239. 10.1161/ATVBAHA.113.303041 24700124 PMC4120754

[B121] XZ S. QH P. XD C. (Editors) (2015). “Study on Distribution Law of TCM Syndromes of Dry Eye,” Proceedings of the 9th National Symposium on Integrated Chinese and Western Medicine Diagnosis (Hunan: Hengyang).

[B122] Yaghoobi G.-H. Shokoohi-Rad S. Jafarzadeh H. Abodollahi E. (2020). Serum Fibroblast Growth Factor 21 in Patients with and without Pterygia. J. Ophthalmic Vis. Res. 15 (1), 38–44. 10.18502/jovr.v15i1.5940 32095207 PMC7001032

[B123] Yang W. Xu H. Yu X. Wang Y. (2015). Association between Retinal Artery Lesions and Nonalcoholic Fatty Liver Disease. Hepatol. Int. 9 (2), 278–282. 10.1007/s12072-015-9607-3 25788195 PMC4387273

[B124] Ye M. Hu D. Tu L. Zhou X. Lu F. Wen B. (2008). Involvement of PI3K/Akt Signaling Pathway in Hepatocyte Growth Factor-Induced Migration of Uveal Melanoma Cells. Invest. Ophthalmol. Vis. Sci. 49 (2), 497–504. 10.1167/iovs.07-0975 18234991

[B125] Ye Q. Wang J. Liu X. Liu Z. BaZong L. Ma J. (2022). The Role of RAD6B and PEDF in Retinal Degeneration. Neuroscience 480, 19–31. 10.1016/j.neuroscience.2021.11.010 34774969

[B126] Yoon K.-H. Lee J.-H. Kim J.-W. Cho J. H. Choi Y.-H. Ko S.-H. (2006). Epidemic Obesity and Type 2 Diabetes in Asia. Lancet 368 (9548), 1681–1688. 10.1016/S0140-6736(06)69703-1 17098087

[B127] Yx L. M L. B Y. (2020). Observation on Clinical Effect of TCM Nursing Acupuncture Therapy on Dry Eye with Deficiency of Liver and Kidney. Chin. J. Ophthalmol. 30 (7), 521–525.

[B128] Zelber-Sagi S. Ivancovsky-Wajcman D. Fliss Isakov N. Webb M. Orenstein D. Shibolet O. (2018). High Red and Processed Meat Consumption Is Associated with Non-Alcoholic Fatty Liver Disease and Insulin Resistance. J. Hepatol. 68 (6), 1239–1246. 10.1016/j.jhep.2018.01.015 29571924

[B129] Zhang M. Li L. Chen J. Li B. Zhan Y. Zhang C. (2019). Presence of Diabetic Retinopathy Is Lower in Type 2 Diabetic Patients with Non-alcoholic Fatty Liver Disease. Medicine 98 (18), e15362. 10.1097/MD.0000000000015362 31045779 PMC6504314

[B130] Zhang X. Zhao L. Hambly B. Bao S. Wang K. (2017). Diabetic Retinopathy: Reversibility of Epigenetic Modifications and New Therapeutic Targets. Cell Biosci 7, 42. 10.1186/s13578-017-0167-1 28815013 PMC5557533

[B131] Zhang Y. Cross S. D. Stanton J. B. Marmorstein A. D. Le Y. Z. Marmorstein L. Y. (2017). Early AMD-like Defects in the RPE and Retinal Degeneration in Aged Mice with RPE-specific Deletion of Atg5 or Atg7. Mol. Vis. 23, 228–241. 28465655 PMC5398883

[B132] Zhong J.-X. Liu L. Sha X.-Y. Wu Y.-N. Chen M.-T. (2020). Lycium Barbarum Polysaccharides Protects Retinal Ganglion Cells against Oxidative Stress Injury. Neural Regen. Res. 15 (8), 1526–1531. 10.4103/1673-5374.274349 31997818 PMC7059572

[B133] Zhou Z.-Y. Wang L. Wang Y.-S. Dou G.-R. (2021). PFKFB3: A Potential Key to Ocular Angiogenesis. Front. Cell Dev. Biol. 9, 628317. 10.3389/fcell.2021.628317 33777937 PMC7991106

